# Dobutamine versus milrinone for inferior ST-segment elevation myocardial infarction with right ventricular involvement: a retrospective cohort study

**DOI:** 10.3389/fphar.2026.1785400

**Published:** 2026-05-15

**Authors:** Si Wang, Qianfeng Xiao, Zhongming Li, Fangyang Huang, Fuxia Lan, Ying Xu, Xin Wei

**Affiliations:** Department of Cardiology, West China Hospital of Sichuan University, Chengdu, Sichuan, China

**Keywords:** cardiogenic shock, dobutamine, inferior STEMI, milrinone, right ventricular myocardial infarction

## Abstract

**Background:**

Inferior ST-segment elevation myocardial infarction (STEMI) complicated by right ventricular involvement (RVMI) leads to right-heart-dominant failure and a higher incidence of cardiogenic shock (CS). Evidence guiding the choice between inotropic agents in this specific phenotype remains limited.

**Methods:**

This single-center retrospective cohort study included 211 patients with inferior STEMI and suspected RVMI treated with either dobutamine (n = 55) or milrinone (n = 156) between June 2009 and June 2025. The primary outcome was 30-day mortality. Secondary outcomes included duration of inotropic support and hospital length of stay. Confounding and missing data were addressed using multiple imputation, multivariable Cox regression, inverse probability of treatment weighting, and sensitivity analyses.

**Results:**

The dobutamine group had more severe hemodynamic and electrical instability, while the milrinone group exhibited greater left ventricular dysfunction. The 30-day mortality rates were 25.5% for dobutamine and 29.5% for milrinone. No significant difference was found in unadjusted (HR 0.863; 95% CI 0.475–1.570; P = 0.632) or multivariable-adjusted Cox analysis (HR 0.612; 95% CI 0.316–1.187; P = 0.153), with results remaining consistent across multiple sensitivity and propensity-based analyses. Notably, among survivors, the duration of inotropic support was significantly shorter in the dobutamine group (3.1 vs. 5.0 days; adjusted β = −1.805; 95% CI −3.147 to −0.463; P = 0.009).

**Conclusion:**

In patients with inferior STEMI and suspected RVMI, no statistically significant difference in 30-day mortality was detected between dobutamine and milrinone, though the study was underpowered to establish equivalence. Dobutamine was associated with a shorter duration of support among survivors despite its application in more unstable patients. Inotrope selection should therefore be individualized based on the patient’s hemodynamic phenotypes.

## Introduction

Inferior ST-segment elevation myocardial infarction (STEMI) accounts for 40%–50% of all STEMI cases, with approximately 30% complicated by right ventricular myocardial infarction (RVMI), particularly in cases with proximal right coronary artery (RCA) occlusion (Goldstein et al., 2024; Nagele et al., 2022; Femia et al., 2021). RVMI impairs right ventricular compliance and cardiac output, resulting in systemic venous congestion and a high incidence of cardiogenic shock (CS) (Goldstein et al., 2024; Nagele et al., 2022; Femia et al., 2021). While isolated inferior STEMI usually carries a favorable prognosis, the superimposition of RVMI and CS dramatically increases short-term mortality, with rates often exceeding 25% (Botti et al., 2025; Josiassen et al., 2021; Jacobs et al., 2003). Compared to left ventricular infarction, RVMI exhibits distinct pathophysiological characteristics. Although timely reperfusion and volume resuscitation are fundamental, the right ventricle has a flat Frank-Starling curve and is highly sensitive to volume overload (Goldstein et al., 2024; Nagele et al., 2022; Femia et al., 2021). Excessive fluid infusion may exacerbate ventricular interdependence *via* septal shifting, compromising left ventricular filling and worsening low-output states (Siniorakis et al., 1994; Dell'Italia et al., 1984). Consequently, when fluid resuscitation is insufficient or limited by congestion, positive inotropic agents become the cornerstone of hemodynamic management.

Selection of the optimal inotrope for RVMI remains controversial. Although dobutamine and milrinone are the most commonly used agents, their comparative efficacy in this setting is unclear. The DOREMI trial found no significant difference in outcomes between the two drugs in general CS patients (Mathew et al., 2021); however, less than half of the participants had myocardial infarction (MI)–related shock, and right-heart dominant cases were underrepresented, limiting generalizability to RVMI (Biswas et al., 2023). Similarly, recent retrospective data on MI-related shock did not specifically stratify by infarct location (Ten Berg et al., 2025). Given the unique dependence of the right ventricle on afterload and the distinct hemodynamic profiles of these agents, a focused analysis is warranted. This study aims to compare the short-term clinical outcomes of dobutamine and milrinone in inferior STEMI patients with evidence of right ventricular involvement, providing real-world evidence to guide personalized treatment strategies.

## Methods

### Study design and population

This single-center retrospective cohort study was conducted at West China Hospital of Sichuan University. We screened adult patients (≥18 years old) admitted between 1 June 2009, and 1 June 2025. Patients were included if they met the following criteria: (1) inferior STEMI confirmed by electrocardiogram (ECG) (ST-segment elevation in leads II, III, and aVF); (2) receipt of dobutamine or milrinone within 7 days of admission; and (3) evidence of right ventricular involvement, defined as a culprit lesion in the right coronary artery (RCA) on angiography or, in the absence of angiographic data, right-sided ECG changes (ST-segment elevation in V3R or V4R). This phenotype-enrichment strategy was designed to identify patients with a high pretest probability of right ventricular involvement rather than confirmed right ventricular dysfunction. Exclusion criteria were: (1) MI occurring >30 days prior to admission; (2) severe mechanical complications (e.g., ventricular septal rupture, free wall rupture, or papillary muscle rupture); (3) MI caused by aortic dissection; (4) concurrent use of both dobutamine and milrinone; and (5) initiation of extracorporeal membrane oxygenation (ECMO). The choice of inotropic agent was not randomized but was made by the treating physician on the basis of clinical judgment. Treatment allocation was therefore influenced by each patient’s hemodynamic phenotype, introducing potential confounding by indication. This was addressed through multivariable adjustment and propensity-based analyes. The study was approved by the Biomedical Ethics Committee of West China Hospital, Sichuan University (Approval No. 2021–1770).

### Data collection and variable definitions

Baseline demographic, clinical, and laboratory data were extracted from the hospital’s electronic medical records system by two independent researchers and validated by a third reviewer. The time of MI onset was defined as the recorded time of symptom onset. Comorbidities, including hypertension and diabetes mellitus, were defined based on discharge diagnoses. The severity of CS was assessed using the Society for Cardiovascular Angiography and Interventions (SCAI) staging system (stages A–E) (Naidu et al., 2022), assigned based on hemodynamic status at the time of hospital admission. CS was defined as reaching SCAI stage C–E at any time during hospitalization. Bradyarrhythmia was defined as clinically significant bradycardia requiring pharmacologic intervention (e.g., atropine or isoproterenol) or temporary pacing. Atrial arrhythmias included atrial tachycardia, atrial flutter, and atrial fibrillation; ventricular arrhythmias included sustained ventricular tachycardia and ventricular fibrillation. Laboratory values were obtained from the initial tests upon admission, and the estimated glomerular filtration rate (eGFR) was calculated using the 2021 CKD-EPI equation. Left ventricular end-diastolic diameter (LVEDD) and left ventricular ejection fraction (LVEF) were obtained from the first transthoracic echocardiogram performed during hospitalization. Coronary angiography was used to identify culprit lesions, assess stenosis severity (≥70%), and determine the presence of multivessel disease. Coronary revascularization was defined as successful percutaneous coronary intervention of the culprit vessel during the index hospitalization. The utilization of inotropic agents, intra-aortic balloon pump (IABP), temporary pacing, continuous renal replacement therapy (CRRT), and mechanical ventilation were ascertained by reviewing medication administration records and procedure notes.

### Study outcomes

The primary outcome was all-cause 30-day mortality, ascertained through telephone follow-up. Secondary outcomes included the duration of positive inotropic support and total hospital length of stay. To minimize survivor bias—whereby early mortality artifactually shortens treatment duration—secondary outcomes were analyzed exclusively in surviving patients. These analyses describe resource utilization patterns among survivors rather than treatment efficacy across the full cohort.

### Sensitivity and subgroup analysis

Sensitivity analyses were performed to assess the robustness of the primary findings: (1) restricting to patients with angiographically confirmed proximal or mid-RCA lesions, and (2) restricting to patients with SCAI stage C–E CS during hospitalization. To further evaluate the impact of the RVMI definition on the primary finding, two *post hoc* analyses were performed using progressively stricter electrocardiographic criteria: patients with both angiographic RCA lesions and any ECG evidence of RV involvement (ST-segment elevation in V3R or V4R, or V1 when right-sided leads were not obtained), and patients with both angiographic RCA lesions and confirmed ST-segment elevation in V3R or V4R. Predefined subgroup analyses were stratified by sex (male vs. female) and the following binary characteristics: age ≥65 years, admission year after 2019, admission <24 h after MI, SCAI stage C–E CS, bradyarrhythmia, eGFR <60, LVEF <50%, proximal or mid-RCA lesions, single-vessel disease, and revascularization.

### Statistical analysis

All statistical analyses were performed using R version 4.3.3 (R Foundation for Statistical Computing, Vienna, Austria). Graphical visualization was performed using Prism 9.0 (GraphPad Software, San Diego, CA, USA). Continuous variables are presented as mean ± standard deviation (SD) and were compared using the Student’s t-test. Categorical variables are presented as frequencies (percentages) and were compared using chi-square tests or Fisher’s exact tests. Kaplan-Meier curves were constructed and compared using the log-rank test. To handle missing baseline data, multiple imputation by chained equations was performed using predictive mean matching (20 imputed datasets, 50 iterations, pooled using Rubin’s rules). Cox proportional hazards models were used to analyze the primary outcome, estimating hazard ratios (HR) and 95% confidence intervals (CIs). The multivariable Cox model (Model 1) was adjusted for baseline covariates, including age, sex, admission lactate, troponin T, serum creatinine, and LVEF. An expanded Cox model (Model 2) additionally incorporated admission era (before/after 2019) and bradyarrhythmia. The number of covariates in these models was constrained by the limited number of outcome events. Inverse probability of treatment weighting (IPTW) was performed using propensity scores estimated from 11 baseline covariates (age, sex, SBP, heart rate, lactate, creatinine, troponin T, LVEF, LVEDD, bradyarrhythmia, and admission era), with stabilized weights trimmed at the 1st and 99th percentiles. For secondary outcomes, multivariable linear regression analysis was conducted, adjusting for the same covariates. Subgroup analyses included interaction terms to assess effect modification. A two-sided P value <0.05 was considered statistically significant.

## Results

### Baseline characteristics of the study population

**FIGURE 1:**
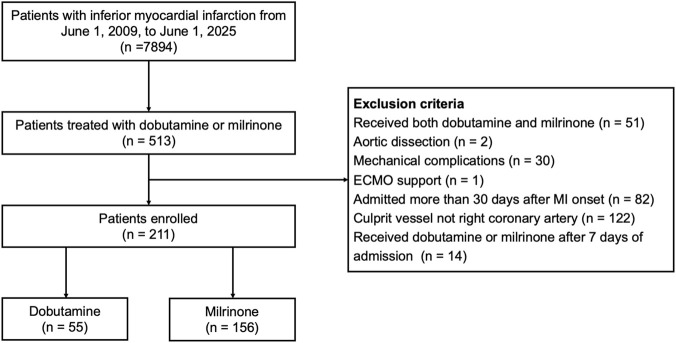
Flow chart of patient selection. Abbreviations: ECMO, extracorporeal membrane oxygenation; MI, myocardial infarction.

A total of 211 patients with inferior STEMI and evidence of right ventricular involvement were included in the study (55 in the dobutamine group and 156 in the milrinone group). The patient selection process is illustrated in Figure 1. Of the included patients, 182 (86.3%) were identified based on angiographically confirmed RCA culprit lesions and 29 (13.7%) based solely on right-sided ECG changes. Baseline demographic characteristics were well-balanced between the two groups (Table 1). However, significant differences were observed in hemodynamic and clinical profiles: the dobutamine group had significantly lower heart rates, smaller LVEDD, and higher LVEF at admission. Furthermore, the incidence of bradyarrhythmias and the requirement for temporary pacemaker implantation were significantly higher in the dobutamine group, which also included a higher proportion of patients admitted after 2019. Subgroup analysis of the 151 surviving patients yielded results consistent with the overall population (Supplementary Table S1). Notably, in this surviving cohort, the dobutamine group had significantly higher lactate and creatinine levels, lower SBP, and higher rates of non-invasive ventilation use, indicating a more severe hemodynamic compromise in this subgroup.

**TABLE 1:** Baseline characteristics of patients in the dobutamine and milrinone groups.

Characteristics	Dobutamine	Milrinone	Total	P value
	(N = 55)	(N = 156)	(N = 211)	
Age, years	69.3 ± 12.8	69.3 ± 12.0	69.3 ± 12.2	0.973
Male	42 (76.4%)	120 (76.9%)	162 (76.8%)	0.933
Height, cm	162.1 ± 7.6	163.2 ± 8.0	162.9 ± 7.9	0.463
Weight, kg	64.3 ± 13.6	64.2 ± 11.1	64.2 ± 11.8	0.948
BMI, kg/m^2^	24.3 ± 4.3	24.0 ± 3.3	24.1 ± 3.6	0.613
Admission year after 2019	40 (72.7%)	68 (43.6%)	108 (51.2%)	<0.001
MI to admission time, days	2.1 ± 3.6	2.0 ± 3.1	2.0 ± 3.2	0.737
Admission <24 h after MI	27 (49.1%)	77 (49.4%)	104 (49.3%)	0.973
SBP, mmHg	105 ± 21	111 ± 23	110 ± 23	0.075
DBP, mmHg	65 ± 17	68 ± 17	68 ± 17	0.261
Heart rate, beats/min	78 ± 26	86 ± 25	84 ± 25	0.041
Hypertension	31 (56.4%)	88 (56.4%)	119 (56.4%)	0.995
Diabetes mellitus	27 (49.1%)	73 (46.8%)	100 (47.4%)	0.769
SCAI stage	​	​	​	0.162
Stage A	4 (7.3%)	28 (17.9%)	32 (15.2%)	​
Stage B	16 (29.1%)	36 (23.1%)	52 (24.6%)	​
Stage C	33 (60.0%)	89 (57.1%)	122 (57.8%)	​
Stage D	1 (1.8%)	3 (1.9%)	4 (1.9%)	​
Stage E	1 (1.8%)	0 (0.0%)	1 (0.5%)	​
Cardiogenic shock	47 (85.5%)	121 (77.6%)	168 (79.6%)	0.212
Bradyarrhythmias	32 (58.2%)	42 (26.9%)	74 (35.1%)	<0.001
Atrial arrhythmias	17 (30.9%)	45 (28.8%)	62 (29.4%)	0.773
Ventricular arrhythmias	11 (20.0%)	19 (12.2%)	30 (14.2%)	0.153
CK-MB, ng/mL	64.9 ± 89.4	85.9 ± 100.6	80.5 ± 98.1	0.176
Troponin-T, ng/mL	2,775 ± 2,995	3,764 ± 3,438	3,509 ± 3,350	0.061
NT-proBNP, ng/L	5,995 ± 8,754	7,531 ± 9,016	7,136 ± 8,954	0.278
Glucose, mmol/L	12.7 ± 8.6	12.0 ± 6.2	12.2 ± 6.9	0.557
Lactate, mmol/L	4.8 ± 4.5	3.7 ± 3.3	4.0 ± 3.7	0.095
Uric acid, μmol/L	449 ± 131	499 ± 307	486 ± 273	0.248
Creatinine, μmol/L	168 ± 128	142 ± 75	148 ± 92	0.070
eGFR, ml/min/1.73m^2^	51.2 ± 28.1	54.4 ± 24.5	53.6 ± 25.4	0.424
ALT, u/l	164 ± 459	182 ± 507	178 ± 494	0.948
AST, u/l	349 ± 1,143	358 ± 793	356 ± 894	0.948
LVEDD, mm	49.1 ± 6.4	51.8 ± 7.3	51.1 ± 7.2	0.019
LVEF, %	49.5 ± 13.9	45.1 ± 13.4	46.2 ± 13.6	0.049
LVEF <50%	24 (47.1%)	91 (60.3%)	115 (56.9%)	0.100
Coronary angiography	48 (87.3%)	134 (85.9%)	182 (86.3%)	0.799
Proximal or Mid-RCA lesion	44 (80.0%)	110 (70.5%)	154 (73.0%)	0.173
Revascularization	45 (81.8%)	121 (77.6%)	166 (78.7%)	0.508
Number of coronary lesions	2.0 ± 0.8	2.1 ± 0.8	2.1 ± 0.8	0.359
Single-vessel lesions	15 (31.3%)	36 (26.9%)	51 (28.0%)	0.562
IABP	15 (27.3%)	38 (24.4%)	53 (25.1%)	0.688
Temporary pacing	25 (45.5%)	28 (17.9%)	53 (25.1%)	<0.001
CRRT	7 (12.7%)	9 (5.8%)	16 (7.6%)	0.094
Invasive ventilation	18 (32.7%)	52 (33.3%)	70 (33.2%)	0.935
Non-invasive ventilation	27 (49.1%)	57 (36.5%)	84 (39.8%)	0.102

Data are presented as mean ± SD, for continuous variables; categorical variables were reported as numbers and percentages (%).

Abbreviations: BMI, body mass index; MI, myocardial infarction; SBP, systolic blood pressure; DBP, diastolic blood pressure; SCAI, society for cardiovascular angiography and interventions; CK-MB, creatine kinase-MB; NT-proBNP, N-terminal pro-B-type natriuretic peptide; eGFR, estimated glomerular filtration rate; ALT, alanine transaminase; AST, aspartate transaminase; LVEDD, left ventricular end-diastolic diameter; LVEF, left ventricular ejection fraction; RCA, right coronary artery; IABP, intra-aortic balloon pump; CRRT, continuous renal replacement therapy; SCAI, stage reflects hemodynamic status at admission. Cardiogenic shock was defined as reaching SCAI, stage C–E at any time during hospitalization.

### Primary clinical outcome

**FIGURE 2:**
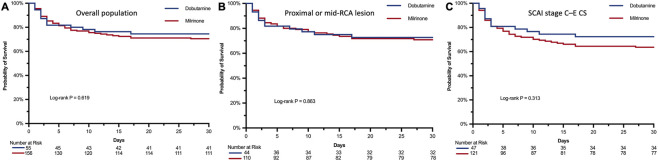
Kaplan-Meier survival curves for 30-day all-cause mortality. **(A)** Overall study population **(B)** Subgroup of patients with proximal or mid-RCA lesions **(C)** Subgroup of patients with SCAI stage C–E CS. Abbreviations: CS, cardiogenic shock; RCA, right coronary artery; SCAI, Society for Cardiovascular Angiography and Interventions.

The primary outcome occurred in 60 patients (28.4%) overall, including 14 (25.5%) in the dobutamine group and 46 (29.5%) in the milrinone group. There was no statistically significant difference in 30-day all-cause mortality between the two groups (P = 0.569). Kaplan-Meier survival analysis demonstrated no significant difference in the cumulative incidence of the primary outcome between the two groups in the overall population (Figure 2A). This finding was consistent in the subgroup of patients with proximal or mid-RCA lesions (n = 154; Figure 2B) and the subgroup with SCAI stage C–E CS (n = 168; Figure 2C).

Cox proportional hazards analysis further quantified these findings. The unadjusted HR for dobutamine *versus* milrinone was 0.863 (95% CI: 0.475–1.570; P = 0.632). After multivariable adjustment (age, sex, admission lactate, troponin T, serum creatinine, and LVEF; Model 1), the adjusted HR was 0.612 (95% CI: 0.316–1.187; P = 0.153), indicating no statistically significant difference in mortality risk between the two groups. In the expanded model additionally adjusted for admission era and bradyarrhythmia (Model 2), the HR was 0.603 (95% CI: 0.298–1.221; P = 0.167). Sensitivity analyses using Model one confirmed the robustness of these results. Restricting the analysis to the proximal or mid-RCA lesion subgroup yielded an adjusted HR of 0.675 (95% CI: 0.309–1.475; P = 0.332). Similarly, in the SCAI stage C–E CS subgroup, the adjusted HR was 0.546 (95% CI: 0.263–1.132; P = 0.111), with no significant between-group difference (Table 2). IPTW analysis yielded a consistent HR of 0.729 (95% CI: 0.360–1.476; P = 0.380). Two *post hoc* sensitivity analyses using stricter RVMI definitions also confirmed the primary finding: restricting to 141 patients meeting both angiographic and ECG criteria yielded an adjusted HR of 0.676 (95% CI: 0.298–1.536; P = 0.358), and further restricting to 86 patients with confirmed right precordial ST-elevation yielded an adjusted HR of 0.783 (95% CI: 0.274–2.241; P = 0.658).

**TABLE 2:** Cox proportional hazards models for 30-day all-cause mortality (Dobutamine vs. Milrinone).

Model	Hazard ratio (HR)	95% CI	P value
Unadjusted model	0.863	0.475–1.570	0.632
Model 1*	0.612	0.316–1.187	0.153
Model 2^†^	0.603	0.298–1.221	0.167

Sensitivity analyses (model 1)	​	​	​
Proximal or Mid-RCA lesions (n = 154)	0.675	0.309–1.475	0.332
SCAI stage C–E CS (n = 168)	0.546	0.263–1.132	0.111

Milrinone was used as the reference group.

* Model one adjusted for age, sex, admission lactate, troponin T, serum creatinine, and left ventricular ejection fraction (LVEF).

^†^ Model two additionally adjusted for admission era (before/after 2019) and bradyarrhythmia.

Abbreviations: HR, hazard ratio; CI, confidence interval; RCA, right coronary artery; SCAI, society for cardiovascular angiography and interventions; CS, cardiogenic shock.

### Subgroup analysis

**FIGURE 3:**
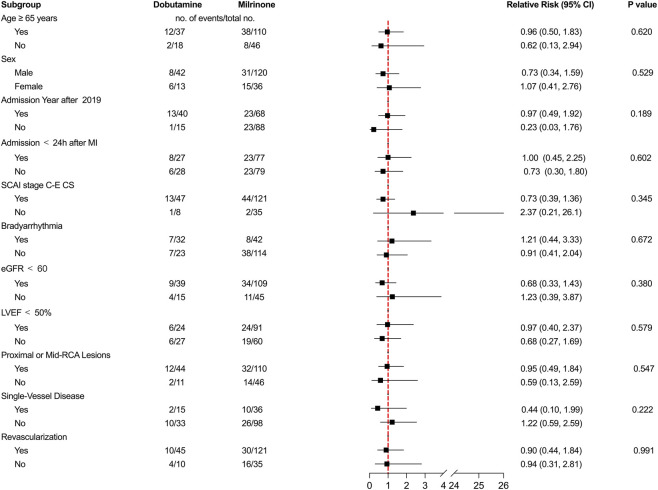
Forest plot of subgroup analysis for the primary outcome. Hazard ratios and 95% CIs for 30-day all-cause mortality comparing dobutamine *versus* milrinone are shown across predefined subgroups. Abbreviations: CI, confidence interval; MI, myocardial infarction; SCAI, Society for Cardiovascular Angiography and Interventions; CS, cardiogenic shock; eGFR, estimated glomerular filtration rate; LVEF, left ventricular ejection fraction; RCA, right coronary artery.

Subgroup analyses were performed across 11 predefined stratification variables. As shown in the forest plot (Figure 3), the risk of the primary outcome did not differ significantly between the dobutamine and milrinone groups in any subgroup. No significant interactions were observed, indicating that the treatment effect was consistent across all specified clinical profiles.

### Secondary outcomes comparison

**FIGURE 4:**
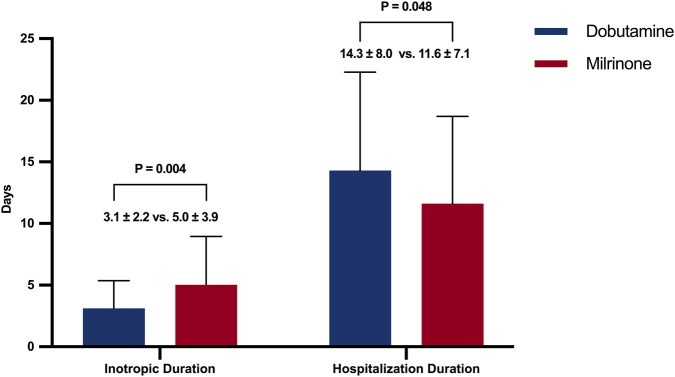
Comparison of secondary outcomes in surviving patients. The duration of inotropic support and total hospital length of stay between the dobutamine and milrinone groups.

Secondary outcomes were analyzed in the 151 surviving patients to avoid artifactually shortened treatment durations due to early death (survivor bias). The analysis focused on the duration of positive inotropic support and total hospital length of stay, presented as resource utilization patterns among survivors. In the unadjusted comparison, the duration of inotropic support was significantly shorter in the dobutamine group compared to the milrinone group, whereas the total hospital stay was slightly longer, though not statistically significant (Figure 4). Dobutamine use was significantly associated with a shorter duration of inotropic support in both univariable (β = −1.914; 95% CI: −3.186 to −0.643; P = 0.004) and multivariable analyses (Model 1: β = −1.805; 95% CI: −3.147 to −0.463; P = 0.009; Model 2: β = −1.779; 95% CI: −3.173 to −0.385; P = 0.014). Hospital length of stay was longer in univariable analysis (β = 2.684; 95% CI: 0.048 to 5.319; P = 0.048) but did not reach significance after adjustment (Model 1: β = 1.732; 95% CI: −0.989 to 4.453; P = 0.214; Model 2: β = 1.814; 95% CI: −0.937 to 4.565; P = 0.198) (Table 3).

**TABLE 3:** Linear regression analysis for secondary outcomes in surviving patients.

Outcome/model	Coefficient (β)	95% CI	P value
Duration of inotropic support			
Unadjusted	−1.914	−3.186 to −0.643	0.004
Model 1*	−1.805	−3.147 to −0.463	0.009
Model 2^†^	−1.779	−3.173 to −0.385	0.014
Hospital length of stay			
Unadjusted	2.684	0.048 to 5.319	0.048
Model 1*	1.732	−0.989 to 4.453	0.214
Model 2^†^	1.814	−0.937 to 4.565	0.198

Coefficient (β) represents the estimated difference in days comparing the Dobutamine group to the Milrinone group (reference). A negative value indicates a shorter duration in the Dobutamine group.

* Model one adjusted for age, sex, admission lactate, troponin T, serum creatinine, and left ventricular ejection fraction (LVEF).

^†^ Model two additionally adjusted for admission era (before/after 2019) and bradyarrhythmia.

Abbreviations: CI: confidence interval.

## Discussion

This study focused on patients with inferior STEMI complicated by suspected right ventricular involvement, a high-risk group that has been historically underrepresented in clinical trials (Goldstein et al., 2024). Our analysis yielded three main findings. First, no statistically significant difference was detected in 30-day all-cause mortality between dobutamine and milrinone, though the study was not powered to establish equivalence. Second, the choice of inotropic agent was largely driven by distinct hemodynamic phenotypes: dobutamine was preferentially used in patients with severe hemodynamic instability and electrical disturbances (“shock phenotype”), whereas milrinone was favored in those with more pronounced left ventricular structural dysfunction (“congestive phenotype”). Third, despite being utilized in patients with a more unstable baseline profile, dobutamine was independently associated with a significantly shorter duration of inotropic support compared to milrinone among survivors.

Our mortality findings are consistent with those of the landmark DOREMI trial and a recent retrospective analysis, which reported no significant difference in survival between the two agents in broader CS populations (Mathew et al., 2021; Ten Berg et al., 2025). However, the DOREMI trial included a heterogeneous mix of CS etiologies, with right-heart dominant cases being a minority, limiting its direct applicability to the specific physiology of right ventricular infarction. To our knowledge, this is the first study to specifically target this population using phenotype-enriched data. Our findings are consistent with the hypothesis that in this setting, neither agent offers a readily demonstrable survival advantage; however, the limited sample size and wide confidence intervals preclude definitive conclusions regarding equivalence, reinforcing the need for nuanced, patient-specific selection rather than a uniform first-line choice.

To interpret these findings, it is crucial to understand the distinct pathophysiology of RVMI. Right ventricular ischemic injury not only impairs systolic function but also significantly affects diastolic filling, leading to increased right atrial pressure and inadequate left ventricular preload (Goldstein et al., 2024; Nagele et al., 2022; Femia et al., 2021). Unlike the left ventricle, the right ventricle is highly sensitive to afterload and volume status (Sanz et al., 2019). While fluid resuscitation is the first-line therapy, RVMI patients exhibit highly heterogeneous volume responsiveness. As confirmed by imaging and hemodynamic studies, acute right ventricular dilation occurs within the non-compliant pericardium, causing a leftward shift of the interventricular septum (Popescu et al., 2005; Goldstein et al., 1982). This structural distortion compresses the left ventricle, impairing its diastolic compliance and filling (Popescu et al., 2005). Given the risk that aggressive fluid resuscitation may worsen this ventricular interdependence and cause pulmonary congestion (Siniorakis et al., 1994; Ferrario et al., 1994), positive inotropic agents play a crucial role in restoring forward flow when fluid resuscitation is insufficient (Arrigo et al., 2024).

The choice between dobutamine and milrinone in this setting is often debated based on their distinct pharmacological profiles. Dobutamine, a selective β1-adrenergic agonist, increases myocardial contractility and heart rate *via* the cyclic adenosine monophosphate (cAMP) pathway (Sinha et al., 2025; Fletcher et al., 2024; Ahmad et al., 2024). Its rapid onset and potent chronotropic effects make it theoretically suitable for acute stabilization (Goldstein et al., 1982), though concerns persist regarding increased myocardial oxygen demand in ischemic myocardium (Sinha et al., 2025; Fletcher et al., 2024; Ahmad et al., 2024). In contrast, milrinone acts as a phosphodiesterase-3 inhibitor, preventing cAMP degradation to produce both positive inotropic and vasodilatory effects (Sinha et al., 2025; Fletcher et al., 2024; Kanwar et al., 2022). Theoretically, milrinone offers specific advantages in RVMI by reducing pulmonary vascular resistance and right ventricular afterload, which are critical determinants of right ventricular ejection (Eichhorn et al., 1987). However, the peripheral vasodilatory effect of milrinone carries a risk of systemic hypotension (Lewis et al., 2019), which may limit its utility in patients with profound shock.

Our study suggests that clinicians navigated these mechanistic trade-offs by tailoring therapy to the patient’s specific hemodynamic phenotype. Although we observed no difference in mortality, the baseline characteristics revealed a clinically logical but analytically challenging pattern of confounding by indication. Patients in the dobutamine group presented with a “shock phenotype,” characterized by lower SBP, higher lactate, and a higher incidence of bradyarrhythmias requiring pacing. These features indicate severe acute right ventricular failure with systemic hypoperfusion. In this context, dobutamine’s ability to rapidly augment cardiac output and restore perfusion pressure appears to be advantageous. As demonstrated by Eichhorn et al., dobutamine improves right ventricular systolic performance primarily through direct inotropic recruitment rather than afterload reduction (Eichhorn et al., 1987).

Notably, the finding that dobutamine was associated with a shorter duration of treatment—despite being used in sicker patients—supports its efficacy in rapidly reversing acute hemodynamic decompensation. This observation was limited to survivors and should not be interpreted as a global treatment effect across the full cohort. The right ventricle is known to have a remarkable capacity for recovery once the acute ischemic insult is stabilized and reperfusion is achieved (Goldstein et al., 2024; Nagele et al., 2022; Femia et al., 2021). Dobutamine may serve as an effective bridge to recovery in this acute phase. Conversely, the milrinone group exhibited more pronounced left ventricular structural and functional damage (larger LVEDD, lower LVEF). This suggests a “congestive phenotype” or biventricular failure, where the afterload-reducing and pulmonary vasodilatory effects of milrinone are more beneficial (Kanwar et al., 2022). The absence of a detectable mortality difference may indicate that clinicians successfully matched the drug to the patient’s profile, suggesting that these agents are complementary tools for different clinical scenarios rather than interchangeable options. However, this interpretation should be considered in light of the limited statistical power to detect moderate differences. Because dobutamine was selectively used in sicker patients, any true benefit of dobutamine may be masked by the higher baseline risk in this group, while conversely, any true benefit of milrinone may be obscured by its preferential use in patients with more chronic structural disease.

Renal function is often cited as a determinant for inotrope selection, as milrinone accumulation in renal failure can lead to toxicity. A sub-analysis of the DOREMI trial found milrinone to be associated with better outcomes only in patients without acute kidney injury (Di Santo et al., 2023). However, our study found no interaction between baseline renal function and treatment effect. This suggests that in the context of acute RVMI, hemodynamic factors—such as maintaining right ventricular perfusion pressure—may outweigh pharmacokinetic considerations regarding renal clearance. Additionally, regarding safety, we found no significant difference in the incidence of arrhythmias between the two groups. This contrasts with the conventional view that dobutamine is more arrhythmogenic (Lewis et al., 2019). A plausible explanation is the high prevalence of vagal-mediated sinus bradycardia and atrioventricular block in inferior STEMI (Goldstein et al., 2024; Aguiar Rosa et al., 2018). In this specific population, the chronotropic effect of dobutamine may serve as a therapeutic benefit to maintain cardiac output, rather than a deleterious side effect.

This study has several limitations. First, the retrospective cohort design inherently carries the risk of selection bias. Treatment allocation was determined by clinical judgment, introducing confounding by indication that was addressed but may not be fully eliminated by multivariable adjustment and IPTW. With 60 outcome events, the events-per-variable ratio was limited; the adjusted estimates should therefore be considered exploratory and hypothesis-generating. Second, the inclusion criteria primarily relied on RCA culprit lesions or ECG changes suggestive of RVMI, rather than confirmed right ventricular dysfunction by echocardiographic or hemodynamic parameters. This phenotype-enrichment approach enhances the applicability to real-world settings but reduces pathophysiological specificity. Future studies should incorporate systematic echocardiographic RV functional assessment to enable more precise phenotyping. Third, despite a 16-year study period, the sample size remained relatively small, which may have limit the statistical power to detect clinically meaningful differences. The absence of statistical significance should not be equated with therapeutic equivalence. Finally, as a single-center study, our findings may reflect institutional-specific practices and may not be fully generalizable to other clinical settings.

## Conclusion

In patients with inferior STEMI complicated by suspected right ventricular involvement, no statistically significant difference in 30-day mortality was detected between dobutamine and milrinone, though the study was underpowered to establish equivalence. However, the choice of agent appeared to be driven by distinct clinical phenotypes. Dobutamine was preferentially used in patients with severe hemodynamic instability and bradyarrhythmias, and was associated with a shorter duration of support among survivors, suggesting a potential role in rapid hemodynamic stabilization during the acute phase. Milrinone was favored in patients with concomitant left ventricular dysfunction. These exploratory findings support an individualized treatment strategy based on the patient’s specific hemodynamic profile and warrant confirmation in larger, prospective studies.

## Data Availability

The raw data supporting the conclusions of this article will be made available by the authors, without undue reservation.
