# Supplementary material for: Dobutamine versus milrinone for inferior ST-segment elevation myocardial infarction with right ventricular involvement: a retrospective cohort study
Source: Front Pharmacol. 2026 May 15;17:1785400. doi: 10.3389/fphar.2026.1785400 (PMC13219343; doi:10.3389/fphar.2026.1785400)
Supplement: Supplementary file 1 [file Table1.docx]

**Supplementary Table S1. Baseline characteristics of surviving patients in the dobutamine and milrinone groups**

|  | Dobutamine | Milrinone | Total | P Value |
| --- | --- | --- | --- | --- |
|  | (N = 41) | (N = 110) | (N = 151) |  |
| Age, years | 68.1 ± 13.8 | 67.7 ± 12.3 | 67.8 ± 12.7 | 0.865 |
| Male | 34 (82.9%) | 89 (80.9%) | 123 (81.5%) | 0.777 |
| Height, cm | 163.4 ± 7.4 | 162.6± 7.9 | 162.8 ± 7.7 | 0.633 |
| Weight, kg | 65.8 ± 13.4 | 64.2 ± 11.7 | 64.7 ± 12.2 | 0.557 |
| BMI, kg/cm^2^ | 24.6 ± 4.6 | 24.2 ± 3.7 | 24.3 ±4.0 | 0.661 |
| Admission Year after 2019 | 27 (65.9%) | 45 (40.9%) | 72 (47.7%) | 0.006 |
| MI to admission time, days | 2.2 ± 3.8 | 2.0 ± 3.2 | 2.0 ± 3.4 | 0.684 |
| Admission < 24h after MI | 19 (46.3%) | 54 (49.1%) | 73 (48.3%) | 0.764 |
| SBP, mmHg | 102 ±21 | 112 ±24 | 109 ± 23 | 0.029 |
| DBP, mmHg | 63 ± 18 | 68 ± 17 | 67 ± 18 | 0.088 |
| Heart Rate, beats/min | 73 ± 22 | 84 ± 23 | 81 ± 23 | 0.008 |
| Hypertension | 20 (48.8%) | 63 (57.3%) | 83 (55.0%) | 0.351 |
| Diabetes Mellitus | 20 (48.8%) | 50 (45.5%) | 70 (46.4%) | 0.715 |
| SCAI Stage |  |  |  | 0.194 |
| Stage A | 4 (9.8%) | 25 (22.7%) | 29 (19.2%) |  |
| Stage B | 12 (29.3%) | 26 (23.6%) | 38 (25.2%) |  |
| Stage C | 25 (61.0%) | 59 (53.6%) | 84 (55.6%) |  |
| Stage D–E | 0 (0.0%) | 0 (0.0%) | 0 (0.0%) |  |
| Cardiogenic Shock | 34 (82.9%) | 77 (70.0%) | 111 (73.5%) | 0.109 |
| Bradyarrhythmias | 25 (61.0%) | 34 (30.9%) | 59 (39.1%) | < 0.001 |
| Atrial Arrhythmias | 11 (35.5%) | 29 (26.4%) | 40 (28.4%) | 0.320 |
| Ventricular Arrhythmias | 6 (14.6%) | 9 (8.2%) | 15 (9.9%) | 0.238 |
| CK-MB, ng/ml | 54.4 ± 84.3 | 81.7 ± 100.1 | 74.2 ± 96.5 | 0.123 |
| Troponin-T, ng/ml | 2941 ± 3279 | 3730 ± 3458 | 3515 ± 3417 | 0.208 |
| NT-proBNP, ng/L | 5831 ± 8635 | 6355 ± 7543 | 6213 ± 7828 | 0.716 |
| Glucose, mmol/L | 12.6 ± 9.4 | 11.6 ± 5.4 | 11.9 ± 6.7 | 0.420 |
| Lactate, mmol/L | 4.3 ± 4.0 | 2.8 ± 2.0 | 3.2 ± 2.7 | 0.009 |
| Uric Acid, μmol/L | 448 ± 141 | 455 ± 124 | 453 ± 128 | 0.770 |
| Creatinine, μmol/L | 170 ± 132 | 131 ± 63 | 141 ± 89 | 0.015 |
| eGFR, mL/min/1.73m² | 51.9 ± 28.7 | 58.4 ± 24.1 | 56.7 ± 25.5 | 0.164 |
| ALT, U/L | 197 ± 524 | 173 ± 544 | 180 ± 537 | 0.808 |
| AST, U/L | 402 ± 1308 | 319 ± 771 | 342 ± 945 | 0.636 |
| LVEDD, mm | 49.4 ± 6.8 | 52.4 ± 7.5 | 51.6 ± 7.5 | 0.030 |
| LVEF, % | 50.5 ± 13.4 | 45.0 ± 12.9 | 46.5 ± 13.2 | 0.027 |
| LVEF <50% | 18 (46.2%) | 67 (62.0%) | 85 (57.8%) | 0.085 |
| Coronary Angiography | 36 (87.8%) | 98 (89.1%) | 134 (88.7%) | 0.824 |
| Proximal or Mid-RCA Lesion | 32 (78.0%) | 78 (70.9%) | 110 (72.8%) | 0.380 |
| Revascularization | 35 (85.4%) | 91 (82.7%) | 126 (83.4%) | 0.698 |
| Number of Coronary Lesions | 1.8 ± 0.7 | 2.1 ± 0.8 | 2.0 ± 0.8 | 0.068 |
| Single-Vessel Lesions | 13 (36.1%) | 26 (26.5%) | 39 (29.1%) | 0.279 |
| IABP | 9 (22.0%) | 20 (18.2%) | 29 (19.2%) | 0.601 |
| Temporary Pacing | 18 (43.9%) | 19 (17.3%) | 37 (24.5%) | < 0.001 |
| CRRT | 4 (9.8%) | 5 (64.5%) | 9 (6.0%) | 0.255 |
| Invasive Ventilation | 5 (12.2%) | 14 (12.7%) | 19 (12.6%) | 0.930 |
| Non-invasive Ventilation | 24 (58.5%) | 40 (36.4%) | 64 (42.4%) | 0.014 |

Data are presented as mean±SD for continuous variables; categorical variables were reported as numbers and percentages (%).

Abbreviations: BMI, body mass index; MI, myocardial infarction; SBP, systolic blood pressure; DBP, diastolic blood pressure; SCAI, Society for Cardiovascular Angiography and Interventions; CK-MB, creatine kinase-MB; NT-proBNP, N-terminal pro-B-type natriuretic peptide; eGFR, estimated glomerular filtration rate; ALT, alanine transaminase; AST, aspartate transaminase; LVEDD,  left ventricular end-diastolic diameter; LVEF, left ventricular ejection fraction; RCA, right coronary artery; IABP, intra-aortic balloon pump; CRRT, continuous renal replacement therapy

SCAI stage reflects hemodynamic status at admission. Cardiogenic shock was defined as reaching SCAI stage C–E at any time during hospitalization.
